# Behavioural Tracking and Profiling Studies Involving Objective Data Derived from Online Operators: A Review of the Evidence

**DOI:** 10.1007/s10899-023-10247-6

**Published:** 2023-08-27

**Authors:** Paul Delfabbro, Jonathan Parke, Maris Catania

**Affiliations:** 1 School of Psychology, University of Adelaide, Adelaide, Australia; 2 Sophro Ltd, Newark, UK; 3 Kindred Group, Valletta, Malta

**Keywords:** Online gambling, Behavioural indicators, Risk, Problem gambling, Responsible gambling

## Abstract

Studies involving the analysis of objective data from online operators attempt to address common concerns about biases in self-report research. This paper surveys the progress in this area of research over the last 15 years. The findings highlight many areas of achievement, including: the development of a set of behavioural markers that reliably differentiate variations in gambler risk. Online gamblers can be grouped into clusters based on the intensity and frequency of gambling; behavioural variability; or, signs of over-commitment (e.g., deposit frequency or expenditure patterns). Behavioural indicators have also been successfully used to predict proxies of harm such as self-exclusion or account closures. However, relatively few studies have combined objective data with self-report data to achieve independent validation of the risk-status of gamblers. Evidence also supports the potential value of short-term responsible gambling interventions involving the use of voluntary and mandatory limits, messages and behavioural feedback. Less work has, on the other hand, addressed the comparative risk of different online gambling products. The findings suggest the need for further validation of findings against independent measures of gambling risk; consistent definitions of indicators; a greater focus on the differentiation of product risk; and, on the long-term impact of RG interventions.

## Introduction

A central feature of gambling research is to understand the phenomenon itself. This includes insights into how much time and money people are spending and if they might be experiencing any harm associated with the activity. For the most part, such insights have traditionally been gained from self-report and principally survey-based methodologies. People will be asked a series of questions about the type and frequency of their gambling and be administered a standardised instrument (e.g., the Problem Gambling Severity Index) to determine whether they are displaying any negative consequences or behaviours indicative of gambling-related harm. Such behaviours might include spending more than they can afford to lose, chasing losses, or borrowing money. Methodologies of this nature have been central to epidemiological and public health approaches to gambling research as well as many psychological studies (Heirene, Wang, & Gainsbury, 2022).

Although methodologies of this type have proved useful in providing insights into the general prevalence of gambling, the relationships between variables, and how people are negatively affected (Browne et al., 2016), many limitations are acknowledged. The first principal concern is that such studies are unlikely to provide accurate estimates of gambling behaviour. For example, inaccuracies may arise when people are asked to recall their behaviours across multiple activities and then partition them to certain time periods (e.g., last 12 months). Even more problematically, people appear to find questions about gambling expenditure very difficult to answer (Blaszczynski et al., 2006). A question such as ‘how much did you spend’ could very easily be interpreted in multiple ways (turnover, net expenditure, or the amount taken along to gamble). In addition, people may not be able to engage in the level of mental accounting that enables them to provide accurate estimates of expenditure, particularly if money on gambling comes from multiple sources or is spread across a variety of activities (Schottler Consulting, 2020). Indeed, estimates in studies of both land-based gambling (Productivity Commission, 1999) and online gambling (e.g., Auer and Griffiths, 2017; Braverman et al., 2014; Heirene et al., 2022) show that actual or net gambling expenditure (or the amount lost) can be under-estimated by more than 50%. A second concern relates to sampling. Standard methods used to capture self-report data (e.g., population prevalence studies) are facing increasingly low response rates, difficulties in sampling across land and cellular numbers, and challenges in capturing certain demographic groups who may be reluctant to participate (e.g., minorities, young people, those with poor cellular coverage) (Jackson et al., 2014). Meanwhile, questions have been raised about whether panel samples offered by research companies (e.g., Qualtrics, Prolific, Amazon Turk) may be biased towards certain demographic groups and be difficult to generalise to broader populations (Pickering & Blaszczynski, 2021).

For these reasons, there has been increasing interest in whether it might be possible to obtain more accurate objective gambling data from other sources. Accordingly, in the last 15 years, several research teams have sought to investigate the potential value of objective data obtained from gambling operators; most notably online operators^1^. Such anonymised data-sets involve accurate objective data of the recorded behaviour of online gamblers using a particular platform for a specified time. Data-sets can include brief sign-up information (e.g., some demographics), the number and frequencies of deposits as well as detailed records of how much is spent, won or lost on different gambling activities per stake, session, or across days. The potential of this information was recognised over a decade ago (see Shaffer et al., 2010; Griffiths and Whitty, 2010) as a way to gain accurate insights into actual gambling behaviour and to be able to profile large samples of gamblers while avoiding the methodological issues described above.

Insights from this type of analysis have been seen to have several important benefits. First, such work has the potential to help operators, regulators and policy-makers identify what people are most at risk of gambling harm and how reliable this can be predicted using operator data. For example, it may be possible to generate more definitive data about what percentage of online gamblers are exceeding safe-gambling limits (e.g., Currie et al., 2008; Dowling et al., 2021) in a way that captures populations of gamblers rather than those who agree to respond to online or telephone surveys. A second potential benefit of this research is that it may help to identify products or structural features of activities which appear to have a stronger association with higher risk gambling or harm. A third important area relates to responsible gambling or secondary public health strategies for people who are actively (and often regularly) involved in online gambling (Korn & Shaffer, 1999). If operators are able to identify people who are showing potentially risky patterns of gambling, there may be opportunities for them to use this as the basis for interventions. As a result, a number of researcher and operator research collaborations have developed around the world which have involved investigations into the characteristics of online gambling, the potential relationship between behaviour and harm (e.g., Xuan & Shaffer, 2009; McAuliffe et al., 2022), and the behavioural impact of responsible gambling interventions (e.g., limits, messaging and behavioural feedback) (e.g., Auer et al., 2014, 2022a, b).

## Aims of this Review

In this paper, we examine the extent to which many of these ambitions have been achieved. Our purpose is to review what is currently known from 15 years of studies involving the analysis of objective behavioural data sourced from gambling operators. In this review, we provide insights into the extent to which the existing literature has covered these different areas. For example, we examine what proxy measures have been used to measure harm; the range and coverage of behavioural indicators; and, what areas appear to be most promising for future assessments of product and player risk as based on current evidence. Given the diversity of approaches, predictors and outcomes in this literature, the current value of meta-analytic approaches is likely to be limited. Instead, what we provide here is an overview of the general trends in the research findings as a way to identify what appear to be the most promising lines of enquiry at this point in time.

## Literature Search Strategy

A search was undertaken using Scopus in December 2022 to identify peer reviewed articles relevant to this review. Key words included: “online/ Internet” AND “gambling/ wagering/ sports-betting”. The term “gaming” was excluded to avoid including the video-game literature. A total of 1795 sources were identified using “gambling” AND “online” and this was found to significantly overlap with the results for “gambling” and “Internet”. Further publications were identified through some earlier published reviews; through inspection of secondary sources cited in the most recent (2022) papers; audits of individual Scopus profiles (e.g., Harvard Medical School team); major funding bodies (e.g., *GambleAware* in the UK); and, using Google searches using the same key terms. Only publications published in English were included (nearly all were). To be included, the studies had to involve objective online behavioural data in at least some part of the research rather than self-reported attitudes or knowledge relating to online gambling. Studies that were only self-report surveys, descriptions of methodology without data, theoretical or scoping reviews, or focused on land-based gambling only were excluded. The search resulted in a total of 56 peer reviewed articles and 2 major reports.

## Definition of Online Gambling

Online gambling was defined as any gambling that was undertaken using a gambling operator using Internet services (whether from a PC or mobile device). Nearly all of this was account based and so does not include gambling occurring using decentralised or blockchain technology (likely to be a very small percentage). Activities included online casino games (slots, blackjack, roulette), online poker, online bingo lottery games, and online wagering (sports or race betting).

## Structural Approach to Review

The key information from studies was compiled in a way to enable readers to gain a rapid overview of the principal areas of focus in these studies; the outcomes investigated (e.g., how do they measure harm or higher risk gambling); the behavioural markers that have been used to detect or predict those outcomes; the methodological approaches; and, the principal trends which are emerging across different studies. It is important to note that not all studies necessarily contain a clear differential between independent measures / predictors and outcomes. Many studies (and particularly the earlier ones) are more descriptive and provide profiles, descriptions or cluster analyses of different groups of online gamblers. Another important caveat relates to the breath of the evidence. As will become clear, much of this research is highly concentrated. A number of papers arise from the same operator; often involve the same group of researchers; and, data has tended to be principally sourced from one part of the world (Europe). Finally, as noted by Gainsbury (2011) and Griffiths and Whitty (2010), it is important to be mindful of how much objective analysis of inferred harm has been verified against other sources of data (e.g., the individual risk status of gamblers as based on their self-report).

## Content Overview

The principal details of the studies are set out in Tables 1 and 2. Table 1 summarises the authors, year, data-sources and aims, whereas Table 2 provides an overview of the methodology and findings. There is a brief summary of the analytical approach taken; the principal behavioural variables included in the investigation; and, the main findings.

**Table 1:** Summary of papers and reports using online behavioural tracking data

	Authors	Year	Geographical source	N	Data source	Gambling type	Analytical approach	Aims of investigation	Focus
1	Adami et al.	2013	Italy	530	-Secondary data analysis of Braverman & Shaffer (2012)	Sports betting	-Segmentation analysis of secondary data	-To find predictors of closed accounts.	Individual risk
2	Auer & Griffiths^c^	2013	Norway	5000	-Norwegian online operator data	Lottery, casino, poker	-Tracked impact of voluntary limit in higher intensity online gamblers	-To determine whether voluntary limit setting affected gambling behaviour	Individual risk
3	Auer & Griffiths	2014	Austria	100,000	-Austrian online operator data	Lottery, casino, poker	-Comparison of ranks of total bets and theoretical loss potential	-To compare theoretical loss to bet amounts	Individual risk
4	Auer et al.	2014	Austria	200,000	-Austrian online operator data	Online slot games	-Comparison of sessions that ended after pop-up message at 1000 slot games	-Comparison of distribution of session terminations post pop-up presentation	Individual risk
5	Auer & Griffiths	2015b	Austria	15,216 &1015	-Austrian online operator data	Varied	-Comparisons of time and money pre and post personalised message	-To determine whether feedback on gambling activity influences behaviour	Individual risk
6	Auer & Griffiths	2016	Norway	17,452	-Norsk Tipping online data	Varied	-Comparison of theoretical loss, amount wagered and gross gambling revenue: message exposure vs. no message exposure	-To determine if messages influence gambling behaviour	Individual risk
7	Auer & Griffiths	2017	Norway	1355	-Norsk Tipping online data	Casino games	-Comparison of self-reported loss with objective loss data	-To determine how accurately people estimate their online expenditure	Individual risk/ Product risk
8	Auer et al.	2020	Europe	49,560	-Norsk Tipping online data	Varied	-Comparison of total amount spent over 12 months for those with or without a voluntary spend limit	-To determine if setting a voluntary limit influences gambling expenditure	Individual risk
9	Auer et al.	2021	Europe	175,818	-Online operator data	Varied	-Comparison of gambling activity before and after limit set	-To profile those who do or do not set a voluntary limit	Individual risk
10	Auer & Griffiths	2022a	Holland	2576	-Dutch online operator data	Casino and sports	-Comparison of gambling behaviour pre and post for those who did or did not receive a call or email	-Whether personalised messages affected subsequent gambling behaviour	Individual risk
11	Auer & Griffiths	2022b	Germany	1000	-Data from a survey of 1000 people and associated data from online German operator	Slot games	-Gambling behaviour compared by those who agreed with/ complied with $1000 mandatory monthly limit on expenditure	-Do people support the mandatory limit, gamble after it is reached and is this related to gambling behaviour	Individual risk
12	Auer & Griffiths	2022c	Europe	43,731	-Operator data from multiple countries	Varied	-Session length / number of bets in session as predicted by structural characteristics of products	-Are certain structural characteristics of online games associated with longer sessions (time and/or number of bets)	Product risk
13	Auer & Griffiths	2022d	Britain	2201	-Operator data from single operator	Varied	-Analysis of whether gambling behaviour changes after a mandatory break on the same or subsequent day	-Does a 60 min mandatory break in play lead to decreased deposits and gambling	Individual risk
14	Auer & Griffiths	2022e	Europe	1287	-PGSI survey data and online data from European operator	Varied	-AI analysis of the relationship between PGSI and measures of within-session gambling intensity	-How do PGs differ from other gamblers in relation to gambling behaviour in sessions and daily activity	Individual risk
15	Auer & Griffiths	2022f	Europe	16,771	-Mentor risk assessment and data from European operator	Varied	-Chasing measure calculated within session; across sessions; across days; sessions with deposits > 1; account depletion used to predict Mentor risk status	-Do chasing measures predict risk status?	Individual risk
16	Braverman & Shaffer	2012	Europe	530	-Online data from a single operator: people who had closed an account	Live-action	-Self reported PG status and expenditure compared with actual operator expenditure	-To determine how accurately people estimate their expenditure and if this is associated with gambling status	Individual risk
17	Braverman et al.	2013	Europe	4056	-Online data from single operator	Casino/ Live action	-Gambling behaviour and other variables used to identify clusters with higher risk gamblers	-What gambling variables predict those flagged by the operator as higher risk	Individual risk
18	Braverman et al.	2014	Europe	2259	-Survey and online data from single operator	Casino/ Live action	-Self-reported PG status and expenditure compared with actual operator expenditure	-To determine how accurately people estimate their expenditure and if this is associated with gambling status	Individual risk
19	Broda et al.	2008	US	47,000	-Data from a single operator	Varied	-Comparison of the gambling behaviour of those who attempt to exceed operator deposit limits vs. those who do not	-Does the gambling behaviour of those who exceed limits differ from those do not exceed limits	Individual risk
20	Brosowski et al.	2012	Europe	27,653	-Secondary analysis of data from single operator	Varied	-Models that examined if engagement in various online activities is associated with exceeding low risk gambling limits	-What product type is most associated with exceeding low risk gambling limits	Individual/ Product risk
21	Catania & Griffiths	2021	UK	7732	-Data from single online operator	Varied	-Profiled the people who sought voluntary self-exclusion	-Is voluntary self-exclusion a valid proxy for problem gambling	Individual risk
22	Catania & Griffiths	2022	UK	982	-Data from single operator	Varied	-Profiled clusters of online gamblers based on DSM proxy measures	-Can DSM proxy criteria be used to profile gambling risk	Individual risk
23	Challet-Bouju et al.	2020	France	1152	-Data from online lottery operator	Lottery products	-Profiled trajectories of online lottery gamblers	-Can higher risk profiles be identified	Individual risk
24	Chen et al.	2022	Belgium	2713	-Data for single game from single operator	Dice slot game	-Compared 3 measures of chasing before and after loss events for low and high involvement players	-Is there evidence of chasing and is it more marked in higher involvement gamblers	Individual risk
25	Dragicevic et al.	2011	Malta	128,788	-Data from single operator	Casino games	-Compared measures of gambling behaviour 4 months after registration	-Is there evidence for clusters of higher and lower risk gamblers	Individual risk/ Product risk
26	Dragicevic et al.	2015	Europe	1218	-Data from multiple operators	Varied	-Compared self-excluders and comparison group on gambling behaviour measures and demographics	-Do self-excluders have a different profile of gambling behaviour	Individual risk
27	Finkenwirth et al.	2021	Canada	175,526 and 2157	-Data from single operator	Slot and casino games	-Machine learning to classify self-excluders and non-excluders using AUC metrics	-How well do gambling behaviours classify people	Individual risk
28	Forrest & McHale	2022	UK	n.a.	-Account data from 7 operators	Wagering and gaming	-Profiled the characteristics of UK online wagering and gaming activities using stratified sampling	-What are the characteristics of online gambling in UK	Individual risk/ Product risk
29	Gainsbury & Russell	2015	Australia	n.a.	-Data from single operator	Sports and racing	-Description of bet types and win and loss %	-What is the profile of betting on an online wagering platform	Individual risk/ Product risk
30	Gray et al.	2012	Varied	2066	-Data from single operator	Casino, Live action sports, fixed odds	-Discriminant function analysis to identify best predictors of people with RG incident vs. no incident	-What is the gambling profile of people with RG incidents	Individual/ Product risk
31	Gray et al.	2015	Iceland	520	-Data from single operator	Varied	-Descriptive analysis of Icelandic online gamblers and trends	-Describe online gambling activity and does it differ by type of activity	Individual/ Product risk
32	Haefeli et al.	2011	Europe	150 & 150	-Data from single operator	Varied	-Quantitative analysis of email communications from gamblers with operators prior to self-exclusion	-Are there markers of future self-exclusion in communications	Individual risk
33	Haeusler	2016	Europe	2696	-Data from single operator	Varied	-Modelling of the best predictors of self-exclusion vs. no self-exclusion	-Does payment behaviour predict self-exclusion	Individual risk
34	Hopfgartner et al.	2022a	Norway	21, 129	-Data from single operator	Varied	-Examined the effects on different play breaks on subsequent gambling	-Do mandated breaks of different duration affect subsequent gambling	Individual risk
35	Hopfgartner et al.	2022b	Varied	25, 720	-Data from single operator	Varied	-Modelling of the best predictors of self-exclusion	-Can self-exclusion be predicted by gambling, monetary use, and demographics	Individual risk
36	Ivanova et al.	2019	Finland	4328	-Data from single operator	Slot games	-Examined predictors and impacts of voluntary limit vs. no limit	-Does being prompted to set a voluntary limit affect subsequent gambling	Individual risk
37	Kainulainen	2021	Finland	n.a.	-Data from single operator	Horse racing	-Examines relationship between previous win/ loss status and subsequent onset of gambling	-Does winning or losing on a previous day influence subsequent gambling activity	Individual risk
38	LaBrie & Shaffer	2011	Europe	679	-Data from single operator	Sports	-Examines what gambling variables differentiate those who close accounts for problem gambling concerns vs. other reasons	-Do those who close accounts for problem gambling concerns differ in their gambling behaviour	Individual risk
39	LaBrie et al.	2007	Europe	40,499	-Data from single operator	Sports betting	-Profiles fixed odds and live action sports bettors	-What are the characteristics of sports betting	Individual risk
40	LaBrie et al.	2008	Europe	4222	-Data from single operator	Casino games	-Profiles of online casino game players	-What are the characteristics of online casino gambling	Individual risk
41	LaPlante et al.	2009	Europe	3441	-Data from single operator	Casino games	-Profiles the characteristics of online poker players	-What are the characteristics of online poker gambling	Individual risk
42	LaPlante et al.	2014	Europe	1440	-Data from single operator & self-report screening tool	Varied	-Examines whether activity type; No. of activities (breadth) and days active (depth) predicts screening measure	-Is breadth or depth of gambling a better predictor of a screening score for higher risk gambling	Individual/ product risk
43	Louderback et al.	2021	Europe	48,114, 2066, 2255	-Multiple data-sets from single operator;	Varied	-Examines behavioural markers of positive PG screening outcomes; RG interventions and self-exclusion	-Are land-based low-risk gambling limits (with additions) able to predict indicators of higher risk status in multiple data-sets	Individual risk
44	Luquiens et al.	2016	France	14,261	-Data from single operator; self-report survey with PGSI	Poker	-Examines predictors of PGSI scores 5 + using gambling and demographic data	-Can higher risk poker gamblers be differentiated using online gambling data	Individual risk
45	Luquiens et al.	2019	France	4887 & 4451	-Data from a single operator	Poker	-Examines time and money spent by self-excluders over 12 months vs. age/ gender matched controls	-Do self-excluders decrease their time and money spending over time	Individual risk
46	Ma et al.	2014	Europe	42,647	-Secondary data from single operator	Varied	Examined within subject dependences in behaviour over time	-Does previous gambling predict subsequent gambling	Individual risk
47	McAuliffe et al.	2022	Europe	16,087 and earlier data	-Data from a single operator 10 years apart	Sports	Examined if harm indicators predicted higher risk gamblers (e.g., those with account closures)	-What harm indicators best predict higher risk gambling	Individual risk
48	Nelson et al.	2008	Europe	47, 603	-Data from single operator	Sports	Examined the difference between self-limiters and no limit setters in terms of gambling behaviour, activity choice, pre and post behaviour	-Does setting a limit influence subsequent gambling behaviour	Individual/ Product risk
49	Nelson et al.	2022	Europe	32,262	-Data from a single operator	Sports	Examined the clustering of different groups of sports bettors based on behaviour and demographics	-Did the set of clusters show similarities with a similar analysis in a previous decade	Individual risk
50	Percy et al.	2016	Europe	604 and 871	-Data from a single operator	Varied	Tested different machine learning algorithms to predict self-exclusion	-What is the best algorithm to predict exclusions	Individual risk
51	Peres et al.	2021	Portugal	n.a.	-Data from 10 operators	Varied	Examined time-series models of betting patterns to develop distinct clusters	-Are there betting patterns and clusters indicative of higher risk	Individual risk
52	Perrot et al.	2018	France	10,000	-Data from single operator	Lottery	Examined the cluster profile of French lottery gamblers	-Are there higher risk clusters of players	Individual risk
53	Perrot et al.	2022	France	7359 & 5079	-Data from single operator & self-report survey with PGSI	Varied	Examines the predictors of different PGSI scores	-How well can gambling variables predict PGSI cut-off classifications. Does this differ for skill vs. chance-based games	Individual/ Product risk
54	PWC	2017	UK	10,635	-4 Major online operators; 12 months of transaction records and player survey including PGSI	Varied	-Modelled predictors of problem-gambler status (PGSI) using demographics and player data. Top 20% ranked players compared with PGSI classification.	-Ability to identify problem gamblers as based on PGSI (scores 8+)	Individual risk
55	Ukhov et al.	2017	Europe	10,000	-Data from single operator	Sports and Casino games	-Compared the predictive value of indicators for self-exclusion in sports vs. casino game players	-Are some predictors more useful for predictions for one activity class vs. another	Individual/ Product risk
56	Whiteford et al.	2022	Europe	24,761	-Data from single operator	Sports betting	-Examined the nature of in-play betting	-What are the relationships between different measures of in-play betting	Individual/ Product risk
57	Wood & Wohl	2015	Sweden	779 & 779	-Data from a single operator	Varied	-Examined whether users of the Playscan tool influenced subsequent gambling behaviour	-Does a RG tool influence expenditure and does this vary by player risk level	Individual risk
58	Xuan & Shaffer	2009	Europe	226 & 226	-Data from a single operator	Varied	-Examined the trends in gambling predicting an account closure vs. matched controls	-Are there identifiable behavioural markers that might presage impending account closures	Individual risk

**Table 2:** Online behavioural tracking studies: predictors and findings

	Author	Predictor variables/ Manipulation	Key outcomes/ findings
1	Adami et al. (2013)	-Frequency (days active in a month); bets per day; variability of stakes; trajectory or slope of stakes	-One segment with a higher proportion of self-reported PGs had higher frequency, intensity and “sawtooth pattern” of stake variability.
2	Auer and Griffiths (2013)	-Activity type	-Casino, lottery and poker players decreased expenditure after limit. Poker players showed largest decrease in time spent.
3	Auer and Griffiths (2014)	-Activity type	-Theoretical loss and bet amounts do not always correspond, particularly with poker and lottery products.
4	Auer et al. (2014)	-Exposure to pop-up message	-Pop-up exposure associated with spike in the number of sessions which terminated early.
5	Auer et al. (2015a)	-Exposure to personalised messages	-Theoretical loss and play duration decreased more than a matched control group without messages.
6	Auer et al. (2016)	-Exposure to different message types	-Total theoretical loss, gross revenue and total wagered less for those who received messages vs. those with no messages.
7	Auer et al. (2017)	-Comparison of measures of objective and subjective loss; product type; level of gambling activity	-Subjective and objective loss estimates were related (small effect size). Self-report estimates were poorer for higher value players and for games with more frequent outcomes (casino games vs. lottery games).
8	Auer et al. (2020)	-Comparison of expenditure between those with limit and those without; gambling intensity bands	-Expenditure declined more in those who set a voluntary limit and those in the highest gambling intensity band.
9	Auer et al. (2021)	-Comparison of active player activity for those who set limit or did not	-Those who set voluntary limits tended to be more loyal (had more behavioural activity) than those who had not.
10	Auer and Griffiths (2022a)	-Comparison of behavioural changes vs. matched controls. Measures included: total bet; number of deposits; days gambled; time spent post 30 days of receiving personalised message via phone or email	-Deposit amounts; deposit frequency; total bets; time spent gambling decreased post 30 days more for those who received messages. Effect not related to intensity of gambling or type of message modality.
11	Auer and Griffiths (2022b)	-Comparison of gambling behaviour by attitudes towards mandatory limit; ongoing gambling; how often limit reached	-60.5% of people reported reaching the monthly limit at least once; over 50% supported it, but self-reported higher value customers were less likely to agree and were more likely to reach limit and gamble after deposit limit was reached. Operator gambling behaviour not related to self-reported problem gambling.
12	Auer and Griffiths (2022c)	-Session length/ number of bets in session as predicted by structural characteristics and demographics (regression) and then decision tree analysis. Predictors include: event frequency, return-to player (RTP), hit frequency, largest win in session, continuity and related deviation measures	-Structural characteristics explained only 7% of variance in session duration; event frequency or occurrences of large wins appeared to be the best predictors in the models.
13	Auer and Griffiths (2022d)	-Number of deposits and % players wagering pre and post mandatory break	-68% of players made no further deposits and only 45% continued gambling on the day of the break. The impact on subsequent days was less clear.
14	Auer and Griffiths (2022e)	-Spend per day; losses per session; top-ups during sessions; sessions with account depletion compared by PGSI status	-Problem gamblers spent more per day; had higher losses during sessions; topped up more in sessions and were more likely to report depletion of their account in sessions.
15	Auer and Griffiths (2022f)	-Chasing compared across risk levels and used as predictors. Chasing measures based on Spearman correlations to determine within, across session and across day chasing; top-ups in session and account depletion	-Top ups within session was the best predictor of gambling risk and proposed as the best operational definition for chasing losses. Account depletions were more common in lower risk gamblers. Within, across sessions and across day chasing measures did not show any definite positive association with gambling risk level.
16	Braverman and Shaffer (2012)	-Cluster analysis: gambling variables in 1st 4 months; these included: days gambled; intensity (bets/days); standard deviation of bets; slope of bet changes over 1st month	-Highest risk cluster with greatest risk of account closure had higher frequency, intensity, variability of bets and upward slope trajectory for bets.
17	Braverman et al. (2013)	-Cluster analysis: number of activities; active days; number of bets; amount wagered in total; bet trajectory over time; average bet size; variability of bets; weekend gambling; time between registration and 1st deposit	-Best predictors in decision tree analysis were number of activities (3 or more) and variability of bets.
18	Braverman et al. (2014)	-Accuracy of estimating gambling losses compared across gambling status; correlation of self-report and objective expenditure	-Higher inaccuracy associated with higher risk gambling, but not in any particular direction.
19	Broda et al. (2008)	-Gambling behaviour compared across two groups (limit vs. no limit). Measures included: % days active; mean bet size; number of bets per day; loss position	-No differences for active days, but attempted limit exceeders made more bets per active day; larger bets, but generally had more favourable loss positions.
20	Brosowski et al. (2012)	-Logistic regression with exceeding limit of 3–4 times per month and the equivalent of $500CAN per year: types of gambling as predictors	-After controlling for multiple engagement, live action sports and poker were the strongest predictors of exceeding low risk gambling limits.
21	Catania and Griffiths (2021)	-Descriptive profile of voluntary self-excluders; total expenditure prior to account closure	-Voluntary excluders are highly variable: many close accounts in 1st week (50%) and many without gambling. May not be a valid indicator of problem gambling.
22	Catania and Griffiths (2022)	-Cluster profile of people based on DSM-5 proxy measures: active days, total bets, hours gambling, deposit amounts and frequency, changes to RG settings, customer contacts, bonus requests, number of credit cards; trajectories in gambling frequency and intensity	-33% of the sample comprised a higher risk cluster with higher values on the majority of DSM-5 proxy measures
23	Challet-Bouju et al. (2020)	-Growth mixture curve modelling using gambling risk indicators: amount spent, number of days active, deposit patterns indicative of chasing, number of games played, largest single deposit, cumulative net losses, *Playscan* risk classification	-Around 5% of customers showed higher risk: more variety of games played, higher spend, higher % of voluntary exclusions, more evidence of chasing based on deposit patterns.
24	Chen et al. (2022)	-Post win/loss analysis of 3 measures of chasing: stake changes, speed of play changes and decision to stop or continue	-Little evidence of chasing. Players tended to stop after losses; increase stake size after wins, although played faster after losses. Little difference between high and low involvement players.
25	Dragicevic et al. (2011)	-Cluster analysis using frequency of betting, intensity (bet sizes), variability of bets and bet trajectory	-Evidence of higher risk clusters who bet more frequently, at higher intensity and with more variability. Slots and roulette tended to be preferred activities of higher risk cluster.
26	Dragicevic et al. (2015)	-Comparisons of behaviour of those who self-excluded or who did not: total expenditure, days played, time in sessions.	-Little difference between the groups in relation to time and frequency metrics, but self-excluders had higher losses.
27	Finkenwirth et al. (2021)	-Classification of models based on gambling variables such as: total days, sessions, total bets, session length and metrics based on variations and ratios based on these. Also included total losses and different games played in session	-Most useful classification variables were: variability of bets within session; bets per day; and, different games per session.
28	Forrest & McHale (2022)	Total wagered; time spent; time of day; frequency; largest losses and associated created variables	-Activity is highly skewed: Top 1% of bettors contributed 52% of betting volume; top 1% of gaming accounts contributed 50%. Horse racing and football most dominant betting activity; slot games dominate gaming (60%) by revenue followed by casino games (36%). Late night bet levels appear to be significantly higher than at other times.
29	Gainsbury and Russell (2015)	Number and type of bets for sports and racing examined; % win and loss; compared bets in wins, places, multi-bets, exotic bets	-The most common bets were on single winning outcomes (45%), but these had higher loss rates and poorer returns than bets on handicap events. Returns were better on less popular sports. Multi-bets had low win rates.
30	Gy et al. (2012)	Number of bets; total wagered; net loss; days gambled (and metrics based on ratios of these)	-People who self-excluded gambled more often and spent more money. The risk was higher for those who engaged in live action betting vs. fixed odds betting.
31	Gy et al. (2015)	Total amount spent; days gambling; time spent and associated ratios	-Most people spent very little, but 96% of bets lost; betting on European soccer attracted higher expenditure from the top 1% highest value customers.
32	Haefeli et al. (2011)	Type of email communications/ content	-Future self-excluders sent more frequent emails; asked more about account opening.
33	Haeusler (2016)	Active months; number, size and variability of deposits; number and size of withdrawals and reversed withdrawals; relative use of different payment methods; number of payment methods	Self-excluders made larger and more frequent deposits and with variability; made larger and more frequent withdrawals and reversed withdrawals; more likely to use mobile phone billing and to use more payment methods. Best multivariate predictor was mobile phone billing.
34	Hopfgartner et al. (2022a)	Effect of 15 s, 90 s and 15 min breaks on subsequent gambling	-Longer pauses in play followed the 15-minute break.
35	Hopfgartner et al. (2022b)	Key predictors included days active; previous exclusions; number of payment methods; number of deposits and by session; withdrawals; variations in bets	-SE higher if the person had previous exclusions; different payment methods; greater % of play on slots; more deposits and during sessions; more withdrawals and reversed withdrawals; higher variability in betting, but fewer active days.
36	Ivanova et al. (2019)	Group comparison of post limit outcomes	-No significant difference was observed between voluntary limit setters and comparison group on net loss and days gambled in follow-up period.
37	Kainulainen (2021)	Survival analysis of gambling onset after winning and losing days	-The onset of subsequent gambling was 27% slower after losing days than break even days.
38	LaBrie & Shaffer (2011)	Discriminant analysis and cluster analysis groups using number of bets, total wagered, total winnings, active days, bet variability	-Those who closed accounts with problem gambling concerns wagered more and more frequently. Two high risk clusters emerged: one had higher bet variability than the other.
39	LaBrie et al. (2007)	Profile of sports betting; correlations; top 1% profile, number of bets, total wagered, total winnings, active days	-the 1% most financially involved sports bettors played over a longer period of time, had more active betting days, had a greater number of bets, placed larger bets and made larger net losses; however, on average they experienced a higher return to player (i.e., lost a lower percentage of money wagered)
40	LaBrie et al. (2008)	Profile of casino gambling; profile of top 5%. No. bets, total wagered, total winnings, active days	-the 5% most financially involved casino bettors played over a longer period of time, had more active betting days, placed a greater number of bets per day, placed larger bets and had larger net losses; however, on average they experienced a higher return to player (i.e., lost a lower percentage of money wagered)
41	LaPlante et al. (2009)	Profile of online poker players; profile of top 5%; No. bets, total wagered, total winnings, active days; sessions; duration	-the 5% most financially involved poker players gambled over a longer period of time, played more sessions, played more sessions per day, lost more per session and had larger net losses; however, on average they experienced a higher return to player (i.e., lost a lower percentage of money wagered)
42	LaPlante et al. (2014)	Logistic regression models and trend plots: relationship between activity type, number of activities, days active and screening measure classification	-Live action sports was the strongest predictor of a positive screening outcome. Days active had a linear association; breadth was linear but asymptotic beyond 4 activities
43	Louderback et al. (2021)	Logistic regression models used to identify best predictors of higher risk status: number of bets per day; total wagered; days active; % income; bet variability across days	-Income % spent; bet variability and losses per month the more reliable predictors of higher risk status. Frequency and betting trajectory proved less reliable predictors.
44	Luquiens et al. (2016)	Logistic regression predicting PGSI 5 + scores using 30-day data: total deposits; mean theoretical loss; number sessions; 3 deposits in 12 h (compulsion); multi-table use	All listed predictors were significant in the models. Model had low specificity (50%) and reasonable sensitivity (80%).
45	Luquiens et al. (2019)	Analysis of time and group interaction for time and money spent	-Self-excluders maintained lower time and money spent vs. controls over 12 months. Higher intensity gamblers only maintained this for limited? time, but showed a re-emergence of higher expenditure vs. controls.
46	Ma et al.	-Number of bets, total wagered frequency, win/loss outcomes for days	-Net cumulative losses and net cumulative gains were associated with subsequent increases in gambling, whereas immediate losses were associated with a reduction in subsequent gambling.
47	McAuliffe et al. (2022)	Logistic regression models used to predict outcomes. Used 9 categories of harm indicator from SeNet in UK. Spend ratio from norm on losing days; time spent; increase in frequency of play; late night play; deposit frequency; failed deposits; reversed withdrawals; multiple payment methods; number of different credit cards. Each category was rated from 0 (no risk) to 3 (highest risk). Coding was based on arbitrary scoring.	-Most harm indictors were able to predict proxies for high risk or problem gambling. However, most of the indicators were very rare and could not be reliably used to predict harm on a given day. Coding thresholds and the relative importance of different indicators in harm scoring remained an issue for further investigation.
48	Nelson et al. (2008)	Between group and pre-post comparisons examined frequency of gambling; bets/ day; bet sizes; total wagered; % lost	Self-limiters tended to have higher levels of gambling activity. Limits led to a reduction in the frequency and total amount wagered although not the mean bet size. Self-limiters had a higher engagement in live-action betting.
49	Nelson et al. (2022)	Groupings were based on frequency and intensity measures (number of games, frequency, duration, number of bets, total wagered; average bet size; net loss; % lost; the magnitude and frequency of deposit events and withdrawal/ reversed withdrawal events	Results were generally similar with the earlier study. Most sports betters gambled a moderate amount, but there was a small percentage with disproportionately higher levels of engagement.
50	Percy et al. (2016)	Modelling included measures of frequency; intensity; trajectory; duration; and, variability	-Very high classification rates were achieved, particularly using a random forest algorithm. The results highlight the value of using supervised models to predict higher risk players.
51	Peres et al. (2021)	Analysis focused on individual bets and win/loss positions	-Distinct betting clusters identified with inferences about the higher risk status of some clusters.
52	Perrot et al. (2018)	Cluster analysis included demographics; total wagered; No. bets, wagers, deposits; largest daily deposit; chasing; No. games played; bet and frequency variability	-The analysis identified a cluster of 3% of players with significantly higher engagement, particularly in scratch card gambling. Most clusters had low expenditure amounts.
53	Perrot et al. (2022)	Classification models using similar data to the 2018 paper above	-Classification rates for PG vs. NPG generally good, but difficult to detect LR and MR gambling above the base NPG category. Deposit variables stronger risk predictors than general gambling intensity markers (e.g., bet amounts), particularly for skill-based activities. Amount wagered more indicative for chance-based games.
54	Price-Waterhouse Coopers (2017)	-Demographics -Account variables (bet volume, mean bets, night betting, variability in frequency and daily position, deposit frequency)	-87% of non-problem and PGs correctly classified (MR and LR not considered) -73% hit-rate or sensitivity (73% of top 20% risk rated were problem gamblers)
55	Ukhov et al. (2021)	-Multiple gambling intensity and frequency variables; variability; gradient variables; how accessed (e.g., laptop)	-Sports betting self-exclusions best predicted by deposits per day; wagers per day; casino self-exclusions best predicted by volume of expenditure; laptop access.
56	Whiteford et al. (2022)	-Quantile regression examined predictors of the frequency of in-play betting. Predictors included: duration (1st to last day); frequency; total stake; stake variability and per day variables based on other measures	-Not all relationships between indicators and the frequency of in-play bets were linear. Relationships varied depending on the overall level of engagement.
57	Wood and Wohl (2015)	-Examined the amount deposited and wagered before and after *Playscan* was used	-Yellow (at risk) players who used the tool were most likely to decrease their deposit amount and amount wagered. Overall, use of the tool was generally low.
58	Xuan and Shaffer (2009)	-Examined trends in gambling behaviour for online gamblers with and without account closures. Predictors included stake amounts, losses, bet trajectories and bet odds	-Those who closed accounts had placed larger bets and had larger net losses closer to account closure but tended to shift from longer (riskier) to shorter odds (less risky gambles).

Notes: Louderback et al. had quite low AUC %s when identifying limits. Does not really show how well their models predict high risk outcomes; only which variables are the best and most consistent predictors across different risk outcomes. PG = Problem gamblers (PGSI); MR = Moderate risk (PGSI); NPG = Non-problem gamblers (PGSI); RG = responsible gambling

### Broad Area Of Focus: Individual vs. Product Risk

It is clear from Table 1 that individual risk (or the description of individual behaviour) rather than product risk has been the principal focus of most studies. Of the 58 publications, 45 (78%) are principally about individual player risk, 21% discuss individual risk as well as some reference to product variations. Only one paper (Auer & Griffiths, 2022d) appeared to be principally focused on product risk. Thus, the current literature provides greater insights into how to identify riskier players rather than riskier online products.

### Proxy Measures of Harm

Table 1 also indicates that some studies used what could be considered as proxy measures of harm, i.e., whether certain predictor variables seemed to be related to a higher risk outcome. Four main outcomes appear to have been favoured in this context. These include the closure of online accounts (e.g., Adami et al., 2013; Braverman and Shaffer, 2012; LaBrie & Shaffer, 2011; McAuliffe et al., 2022; Xuan and Shaffer, 2009); responsible gambling (RG) incidents of various types (Gray et al., 2012); setting higher gambling limits (e.g., Braverman et al., 2013; Chen et al., 2022); and, whether people exceeded low risk gambling limits set previously for land-based activities (e.g., Braverman et al., 2014; Louderback et al., 2021). Other studies offered profiles of gamblers (e.g., based on cluster or latent class analysis) which attempted to infer that a certain top percentage of customers (e.g., based on a combination of variables) were higher risk and different from the rest of the customer population (e.g., Braverman and Shaffer, 2012; Perrot et al., 2018). A final method has been to use commercial algorithms (e.g., Catania and Griffiths, 2022 report a set of indicators mapped to DSM-5 diagnostic criteria for gambling disorder, whereas Auer and Griffiths, 2022f refer to the *Mentor* system which is a methodology developed by the first author). Wood and Wohl (2015) presented findings relating to *Playscan* developed by *Svenska Spel*.

### Independent Measures of Harm/ Problem Gambling

There were 6 studies which included an independent self-report measure of problem gambling. Some of these used the Problem Gambling Severity Index (PGSI) (e.g., Auer & Griffiths, 2022e; Luquiens et al., 2019; Perrot et al., 2022; Price-Waterhouse Coopers, 2017); others used brief screening measures such as the Brief Psychosocial Gambling Screen (e.g., Braverman et al., 2014; LaPlante et al., 2014). These studies were able to compare the classifications of risk based on behavioural measures with PGSI classifications; one of the best examples of this is the PWC report in the UK which compared the top 20% of “riskiest” players with PGSI scores of 8+ (problem gambling range).

### Responsible Gambling Measures

A number of studies have examined the effectiveness of responsible gambling measures. These include the effects of voluntary limits (e.g., Auer and Griffiths, 2013, 2020, 2021; Ivanova et al., 2019; Nelson et al., 2008); mandatory limits (Auer & Griffiths, 2022d); breaks in play (Auer & Griffiths, 2022d; Hopfgartner et al., 2022a) or the effects of pop-up or personalised messages (Auer et al., 2014; Auer & Griffiths, 2015b, 2016, 2022a). These studies generally employ a pre-post design (sometimes with a comparison group) to determine whether expenditure, time spent, or the number or size of account deposits differ after exposure to the intervention.

### Categories of Behavioural Indicator

Table 2 shows that a range of different behavioural indicators have been studied. For ease of reference, these are summarised into categories into Table 3. The list displayed in Table 3 is not exhaustive because of the capacity to create all manner of calculated variables using machine learning and other algorithmic methods but should provide a comprehensive guide to what information has been used. As Table 3 indicates, one of the most measured aspects of gambling is the intensity or how much people gamble. This can be broken down into 4 main categories: frequency; expenditure level; duration; and, speed. Studies appear to vary in whether some of this information is provided in the form of primary data or calculated from macro-level data such as: total wagered, total sessions, total minutes and days gambled. A particular area of contention worth noting is whether it is better to use theoretical loss as opposed to actual net expenditure when calculating the intensity of gambling. Auer and Griffiths (2014) have used a method which essentially multiplies the house edge by the amount staked to estimate losses, but this has attracted criticism from some authors (e.g., Tom and Shaffer, 2016a, b) on the grounds that it may not generalise to all forms of gambling. For example, people may play a suboptimal strategy on card games such as blackjack, so that their returns are lower than the assumed house edge. The house edge may also be difficult to calculate for some activities such as sports and racing, which are odds-based activities which may not have a mathematically prescribed expected return to players. These points appear valid, although, as Auer and Griffiths (2014) note, the correlation between theoretical loss and actual expenditure may be high enough (e.g., 0.85) to allay concerns about the generalisability of the theoretical loss measure.

A second category relates to the input or output of money from gambling platforms. A number of studies have examined the size and frequency of player deposits into their gambling accounts (Auer & Griffiths, 2022a, d; Haeusler, 2016; Luquiens et al., 2016). Deposits which are greater in size, frequency and variability have been considered potential indicators of greater gambling risk; and this has been considered even more indicative if there is evidence of deposits being rejected (e.g., due to inadequate funds or a suspicion of illegal activity by the operator). Higher risk players may make repeated deposits (i.e., top-up their balances within the same session of play) (Auer & Griffiths, 2022f) which is hypothesised to be a sign of chasing. Withdrawals of money are also considered potential useful indicators of risk if players reverse the action; such behaviour may indicate chasing or an inability to allow winnings (a potential source of funding for further gambling opportunities) to be removed (McAuliffe et al., 2022).

A third category of indicators which can be grouped into different subcategories relates to dynamic changes in behaviour (e.g., Haeusler, 2016). Several studies have examined chasing based on whether people increase their bets following losing outcomes, sessions or days (e.g., Auer and Griffiths, 2022f; Challet-Bouju et al., 2020) or placed bets with longer odds (e.g., Xuan & Shaffer, 2009). Studies have also examined the variability of behaviour (e.g., the amount wagered from day to day) with some (e.g., Adami et al., 2013) referring to a “saw-tooth” pattern in which higher risk was seen to be characterised by a cycle of higher and lower expenditure (either due to chasing losses or a cyclical reduction in expenditure due to inadequate funds). A third category of dynamic variable (e.g., Adami et al., 2013; Braverman and Shaffer, 2012) has involved the calculation of behavioural gradients or slope values based on the change in a given outcome variable (e.g., amount wagered per day) calculated against variations in time. Higher slope values are taken to indicate higher risk gambling.

A fourth category of indicators relates to statistical anomalies in the timing of gambling activity: how often the person gambles late at night (Forrest & McHale, 2022; McAuliffe et al., 2022) or gambling on weekdays rather than weekends (Braverman et al., 2013). The logic here is that people who may have less control over their gambling are engaging in activity during the night either to disguise their gambling from others or because this reflects an inability to stop gambling.

A fifth category (as mentioned above) relates to any activity that might indicate a self-recognised risk. Voluntary exclusions; account closures; or, contacts to customer service about gambling-related problems are all indicators of risk. Other more subtle indicators which have been examined include contacting customer services for other reasons (e.g., to seek bonuses) (e.g., Gray et al., 2012).

A final category which (as indicated above) has been less commonly studied relates to product selection and engagement. Indicators of risk that have been studied include gambling on a wider range of products (often termed “breadth” to distinguish it from the depth of level of engagement, see LaPlante et al., 2014) or on specific products that are considered to be riskier by design. For example, rapid, more continuous products such as in-play sports betting (e.g., Brosowski et al., 2012; Gray et al., 2012) or slots (e.g., Dragicevic et al., 2011; Hopfgartner et al., 2022b) have been reported to be higher risk than products that have more natural breaks in play or which afford fewer opportunities to bet. It has been hypothesised that rapid and continuous games lead to increased losses, poor decision making and may encourage chasing behaviour.

**Table 3:** Categories of behavioural indicator

Indicator category	Examples of higher risk behaviours
Level of gambling intensity / engagement	
Frequency	-Greater number of active days; % of sampled days on which a person gambles; Number of bets; Number of sessions
Duration	-Greater number of hours played; longer session durations; greater number of days spanning first active day to last active day
Expenditure level	-Higher bet sizes; higher net loss; higher theoretical loss; higher total amount wagered
Speed / How fast people play	-Greater number of bets per unit time (e.g., per hour or session)
Money in/ Money out	
On-ramp behaviour (deposits)	-Greater size and frequency of deposits and largest amount deposited; more declined or rejected deposits; larger number of payment methods; greater number of credit cards; source of payment (e.g., mobile vs. PC);
Off-ramp behaviour (withdrawals)	-Frequency of withdrawals or reversed withdrawals
Account depletion	-More likely to finish a session with no funds left in account
Dynamic behaviours / patterns	
Chasing	-Increases in bet size within or across sessions; days; betting at shorter odds; greater frequency of top-ups during session; top-ups increasing in size
Variability	-Greater variability in the size or frequency of bets and/or deposits; gambling frequency (e.g., per week)
Gradients/ slope functions	-Increases in bet size, amount wagered, and/or deposit size or frequency as a function of time
Statistical anomalies	
Time of day	-Higher percentage of play at unusual hours or at night
Time of week	-Higher percentage of play occurring on weekdays rather than weekends
Customer contact	-Higher frequency of interactions with customer service; More frequent requesting of bonuses
Responsible gambling activity	Browsing RG pages; selection of RG tools; changing or removing use of RG tools
Product-based variations	
Breadth of products	-Engaging in a larger number of different categories of product (e.g., slots, table games, poker etc.)
Risky product selection	-Greater engagement in rapid, more continuous products such as slots or in-play sports betting

## Principal Findings: Behavioural Risk Indicators

In this section, we summarise what is generally been found in relation to each of the content areas. We commence with a discussion of the behavioural indicator findings and then provide an overview of findings from responsible gambling studies (Table 3). Inspection of the general pattern of results from studies that included measures of intensity and engagement generally allow several conclusions to be drawn: (a) gambling expenditure on online platforms is highly skewed, with the vast majority of people having low levels of expenditure, but with clearly identifiable higher risk clusters (often comprising 1 to 5%) of the customer population (Catania & Griffiths, 2022) across different product types including, for example, sports betting (LaBrie et al., 2007), casino (LaBrie et al., 2007), and poker (LaPlante et al., 2009). Analysis (e.g., Forrest & McHale, 2022) shows that the top 10% of gamblers on online platform appear to account for 50%+ of total turnover and/ or net revenue; (b) most people lose money, but that returns to player appear to be higher (i.e., a lower percentage of money wagered is lost) in the highest value players; (c) studies using proxy measures of gambling risk show that people who close accounts, who evoke RG interventions or who exclude themselves tend to have a higher engagement with gambling (total amount wagered, frequency of gambling, total sessions and duration of gambling); (d) a significant proportion of people exceed low risk gambling limits (Louderback et al., 2021); (e) Studies using proxy measures have been validated in studies which have also been able to administer the PGSI (Auer & Griffiths, 2022e; Luquiens et al., 2019; PWC, 2017) using associated self-report surveys. These studies generally show higher levels of objective gambling intensity and involvement (frequency, expenditure) in higher risk gamblers as classified by the PGSI.

Support for the second category (Money in/ Money out) has also generally been obtained with more frequent and larger deposits amongst those classified as problem gamblers on the PGSI (Auer & Griffiths, 2022e; Luquiens et al., 2019), and more reversed withdrawals (although some of these behaviours are often so rarely observed as to be less useful in models, see McAuliffe et al., 2022).

Studies of the dynamics of gambling behaviour (Category 3) have generally been more mixed. Some studies have found a positive wagering gradient for those in higher risk clusters (e.g., Braverman et al., 2011) or who subsequently closed an account (Xuan & Shaffer, 2009), while other studies have found little evidence for stake increases following losses within or across sessions (e.g., Auer and Griffiths, 2022f; Chen et al., 2022). However, Ma et al. (2014) found that the scale and immediacy of losses are an important consideration such that, while gambling behaviour may decrease following an immediate loss, it will increase following a longer period of accumulated net loss. Support for chasing behaviour as a risk marker may also depend on how it is operationally defined. When defined as ‘frequent within-session depositing’ this construct receives more consistent support (Auer & Griffiths, 2022f; Challet-Bouju et al., 2020), whereas chasing in the form of making longer odds (i.e., riskier, less probable) bets to recoup losses was not observed by Xuan and Shaffer (2009). In a more nuanced consideration of how chasing might provide a behavioural indication of high-risk gambling, Adami et al. (2013), reported that a “saw-tooth” pattern of behaviour (i.e., a cyclical pattern involving the gradual ‘ramping up’ of staking behaviour followed by a ‘crash’) was more common in higher risk gamblers. Although some players may increase their bets to chase larger wins, this pattern appears more consistent with a pattern of chasing losses given the sudden decrease in staking sizes after the peak is reached, thereby suggesting a decrease in the availability of funds. This observation is consistent with the other studies which found greater variability in behaviour to be indicative of higher risk (e.g., Braverman et al., 2011, 2013).

In relation the second to last category, studies further show that gambling late into the night appears to be generally supported as an indicator of higher risk gambling (e.g., Forrest & McHale, 2022; McAuliffe et al., 2022).

The final category product risk generally shows two main findings: (a) People who gamble on a wider range of products (i.e., greater “breadth”) generally appear to be higher risk gamblers (e.g., Braverman et al., 2013; LaPlante et al., 2014); and, (b) have greater involvement in faster, more continuous activities such as slot games (Dragicevic et al., 2011; Hopfgartner et al., 2022b) or in-play sports betting. In general, however, relatively little research has been undertaken to examine how indicators of behavioural risk can be used to identify higher risk products or structural characteristics that might encourage riskier styles of play (e.g., greater variability in betting or chasing). In one of the few exceptionsAuer and Griffiths (2022c) found that many different behavioural variables were significantly associated with increases in bets per session and theoretical loss, but this study did not include any proxy (e.g., exclusion or account closure data) or direct measures of problem gambling or harm (e.g., PGSI).

## Principal Findings: Responsible Gambling Features

Most of the studies of RG involve the Auer and Griffiths collaboration. As shown in Table 2, the principal RG categories include: voluntary limits; mandatory limits; pop-ups; messaging; breaks-in-play and voluntary exclusion. All of the studies involving voluntary self-exclusion showed that those who excluded tended to decrease their gambling intensity on at least one measure (whether expenditure, time spent or other measure) (Auer & Griffiths, 2013; Auer et al., 2020, 2021; Broda et al., 2008). Mandatory breaks also appear to lead to decreases in gambling on the same day (Auer & Griffiths, 2022d) (60-minute break) or where the limit was set at 15 min rather than shorter intervals (Hopfgartner et al., 2022a). Pop-up messages were found to lead to increases in the number of sessions terminated early (Auer et al., 2014), personalised communications or feedback (e.g., spend relative to limit) have been associated with subsequent decreases in gambling (Auer & Griffiths, 2022a; Auer et al., 2015a, 2016). Voluntary exclusion has also been associated with reductions in gambling (Catania & Griffiths, 2021; Dragicevic et al., 2015); however, as Catania and Griffiths (2021) caution, such behaviour can sometimes be observed very soon after registration by people who do not gamble at all.

## General Discussion

Taken as a whole, this body of work provides many important insights into the nature of online gambling. Expenditure is generally highly concentrated in a very small percentage of participants; most people lose money, but also generally do not spend large amounts. Using various statistical techniques (e.g., cluster, latent class, or machine learning / decision-tree approaches), it is possible to identify a small percentage of higher risk gamblers whose gambling (at least) suggests that they consistently exceed safe gambling limits (e.g., Currie et al., 2008; Dowling et al., 2021) and who are statistically much more likely to show evidence of harm. In a number of studies, this has been inferred through a higher prevalence of proxy measures of harm (e.g., self-exclusions and account closures). A small number of studies have also shown that groups identified as higher risk based on their objective data score in the problematic range on the PGSI (e.g., PWC, 2017). Studies show that these high-risk clusters can be identified based on a range of risk indicators. These extend beyond indicators of gambling intensity (frequency, time and expenditure level) to encompass: differences in depositing behaviour; whether people reverse withdrawals; when people gamble; the range of activities they choose; and, also if they interact with the operator in a way that indicates signs of harm or escalating involvement (e.g., requests to reduce RG limits or obtain additional bonus opportunities). Importantly, the research shows that there may be important dynamic changes which are observable over time which are indicative of higher risk gambling. These include variability in several measures of gambling involvement, the most notable of which may be upward slope trajectories in staking, expenditure and deposit patterns. Such variability in the form of upward slope trajectories may be followed by a steep downward slope or ‘crash’ indicating that further gambling may no longer be economically sustainable (e.g., Adami et al., 2013). Exactly to what extent this variability reflects loss-chasing may depend on how loss-chasing behaviour is defined and over what time scale it is measured.

All of these findings provide an important evidence base that can help support and inform attempts to develop regulatory and industry standards relating to risk detection for online operators. However, it is evident that some decisions would need to be made about how this information can be used to meet the needs of operators and regulators. Findings that are based upon academic research are often retrospective and based on variable coding, offline statistical analysis, and several layers of decision-making often driven by earlier stages of the data-analysis. As a result, it may not always be available in real-time. Accordingly, for researchers who work directly with operators, the answer has been to adopt a more algorithmic or machine-learning approach that tries to capture real-time variables. Examples of this approach are summarised in Percy et al. (2016), Wood and Wohl (2015) and Catania and Griffiths (2022).

Machine-learning and algorithmic approaches differ from more theoretically driven academic approaches in that they are more specifically focused on prediction. All manner of variables (e.g., complex interactions) may be included in models to achieve the best possible predictions and the meaning of these may not always be immediately evident in a theoretical sense (e.g., late night play on a weekday x 5 + wager per hour x increasing bet slope). Such predictive methods may be effective in classifying high risk gamblers, but may not be easy to scrutinise and evaluate. Many of these algorithms may not necessarily use the set of standard risk variables (usually an accumulation of single and definable variables) that academic research has suggested. Thus, an important issue for discussion in this area is whether these are complementary or divergent approaches to the same research problem. If commercial operators are classifying risky gamblers using their own custom algorithms, will these be accepted by the broader academic community or be treated with suspicion if they are difficult to understand?

## Limitations of the Existing Evidence Base

Our review also underscores many the gaps and areas where caution needs to be expressed about objective behavioural analyses. Independent validations of objective risk against self-reported risk (e.g., PGSI scores) are rare (e.g., PWC, 2017). Although the studies that have included these validations show promising results, models tend to be unsatisfactory unless they are comparing people classified as problem gamblers vs. non-problem gamblers. Detecting lower or moderate risk gambling and differentiating it from non-problem gambling is harder and this potentially limits the public health benefits of models if the interest is on minimising harm and preventing it. Nevertheless, as Wood and Wohl (2015) show, this does not prevent the use of more graded RG systems that provide interventions and information (e.g., yellow light, red light) to capture the varying levels of risk that might apply to different customers.

A second and related limitation is that few studies have examined how variations in objective behaviour are related to independently assessed measures of harm. Although a number of studies have examined proxy measures of harm (e.g., account closures, declined deposits) or requests for assistance, it would be good to strengthen this work by examining whether people who display certain patterns of behaviour report greater harm associated with gambling, e.g., financial, psychological or in their social relationships.

A third limitation that needs to be addressed is product risk. The vast majority of papers are designed to assist in the identification of higher risk individuals, but operators and regulators may also need guidance in which products and game features need greater attention. Are there populations of product users who should receive greater RG attention, and should regulators be more wary about the expansion or introduction of certain online products and features rather than others? The assessment of product risk is a different research question and may require a focus on different range of risk indicators or, a different emphasise. For example, while individual risk assessments often focus on how people gamble (time, money, variations), this may not necessarily indicate greater product risk. Some activities may be relatively cheap to play at a population level (e.g., slot games), but have a higher risk of harmful play. Thus, the average expenditure per session or player may not always indicate higher product risk per se. For these reasons, studies of product risk suggest that other factors need to be considered. These include: the speed of the game; (Delfabbro et al., 2020); the timing of bets (e.g., in-play vs. pre-match) (Hing et al., 2019); accessibility; the accessibility of activities; the role of promotions and inducements; and, the range of activities utilised (Gainsbury, Angus, & Blaszczynski, 2019).

Third, the review also shows how there can be further score for the expansion of the range of indicators used to identify higher risk individual gambling. So far, much of the focus on assessing the risk of products has been based on whether engagement in certain products is associated with greater gambling intensity (e.g., number of bets or theoretical loss) (Auer & Griffiths, 2022b) or exceeding low-risk gambling limits (Brosowski et al., 2012), but such work could be expanded to include a wider range of indictors, even when markers are harder to define and measure (e.g., chasing), or are less frequently observed in the population of players (e.g., declined deposits) as these atypical indicators may prove useful for grading risk at the more extreme end of the spectrum.

Fourth, in conducing this review, we acknowledge the broader limitations of objective data studies: sample representativeness, account usage, and, competing activities. Those who gamble online are usually more likely to be male, younger, highly educated or have higher incomes than other gamblers (see Gainsbury, 2012; Hing et al., 2022), so that findings cannot be generalised to other land-based forms of gambling. Studies can also not control for shared use of account, the use of accounts by different operators. Moreover, without self-report survey data, one does not know if people’s problems may be, at least in part, caused by concomitant land-based gambling activities.

Finally, it should be acknowledged that research in this area is likely to be improved through greater collaboration and transparency in the sharing of objective data, analytical techniques and replication of results across different gambling markets, countries and methodological approaches. For example, greater convergence of findings and advancement of insights may result from the development of consistent definitions of key variables (e.g., chasing), sharing of methodological approaches and data, and collaboration between researchers, industry groups and regulators (Delfabbro et al., 2021).

## Conclusion

In conclusion, our review shows that considerable progress has been in online gambling research. Objective data-analysis has an important place in the field of gambling studies and shows considerable potential as a way to capture the behaviour of online gamblers, different levels of risk, and the potential of online responsible gambling initiatives. Important future developments include the further consolidation and standardisation of behavioural indicators, including their definition and measurement, as well as the independent validation of risk models against other sources of data that captures the use of other online operators as well as engagement in land-based gambling. It is also hoped that additional work will be undertaken to understand how indicators can be used to profile the risk of different gambling products to provide regulatory guidance on what products may be “safer” or riskier for players.

## Footnotes

1 The authors acknowledge that there are many studies which have gained access to data from land-based operators (e.g., in the UK and Australia). However, the principal focus of this paper is on online data analysis.

## Declarations

### Jonathan Parke

Jonathan has received support for research, travel and speaking engagements from a variety of government and non-government sources including AGRI and GambleAware. He has conducted a number of commissioned reports for industry groups. The principal focus of this work has been on harm minimisation, responsible gambling and risk associated with different gambling products and features.

### Maris Catania

Maris has been employed by the Kindred Group in roles involving research, player protection, risk assessment and responsible gambling.
